# Response to clozapine in treatment resistant schizophrenia is related to alterations in regional cerebral blood flow

**DOI:** 10.1038/s41537-024-00544-3

**Published:** 2024-12-23

**Authors:** Junyu Sun, Fernando Zelaya, Kyra-Verena Sendt, Grant McQueen, Amy L. Gillespie, John Lally, Oliver D. Howes, Gareth J. Barker, Philip McGuire, James H. MacCabe, Alice Egerton

**Affiliations:** 1 Department of Psychosis Studies, Institute of Psychiatry, Psychology & Neuroscience, King’s College London, London, UK; 2 Department of Neuroimaging, Institute of Psychiatry, Psychology & Neuroscience, King’s College London, London, UK; 3 Department of Psychiatry, Warneford Hospital, University of Oxford, Oxford, UK; 4 Department of Psychiatry, University College Dublin, Dublin, Ireland; 5 Department of Psychiatry, St Vincent’s Hospital Fairview, Dublin, Ireland; 6 National Psychosis Unit, South London and Maudsley NHS Foundation Trust, London, UK

**Keywords:** Schizophrenia, Pharmacology

## Abstract

PET and SPECT studies in treatment-resistant schizophrenia (TRS) have revealed significant alterations in regional cerebral blood flow (CBF) during clozapine treatment, which may vary according to the clinical response. Here, we used the more recent MRI approach of arterial spin labelling (ASL) to evaluate regional CBF in participants with TRS (*N* = 36) before starting treatment with clozapine compared to in healthy volunteers (*N* = 16). We then compared CBF in the TRS group, before and after 12 weeks of treatment with clozapine (*N* = 24); and examined the relationship of those differences against changes in Positive and Negative Syndrome Scale for Schizophrenia (PANSS) scores over the treatment period. We observed widespread reductions in CBF in TRS compared to in healthy volunteers (*p* < 0.05). After covarying for global CBF and age, lower CBF in frontal and parietal regions was still evident (*p* < 0.05, FWE corrected). Clozapine treatment was associated with longitudinal decreases in CBF in the anterior cingulate cortex (ACC) (*p* < 0.05). Higher striatal CBF at baseline was associated with greater improvement in total and general symptoms following clozapine, and higher hippocampal CBF was associated with greater improvement in total and positive symptoms. Longitudinal reductions in CBF in the ACC and thalamus were associated with less improvement in negative (ACC), positive (thalamus), and total (thalamus) symptoms. These findings suggest that changes in CBF on clozapine administration in TRS may accompany symptomatic improvement, and that CBF prior to clozapine initiation may determine the degree of clinical response.

## Introduction

Around one-third of patients with schizophrenia show treatment resistance^1,2^. Clozapine is the only recommended antipsychotic for treatment-resistant schizophrenia (TRS), and ~50% of those with TRS will respond to clozapine^3,4^. Neurobiological theories of TRS compared to treatment-responsive illness include dopaminergic, glutamatergic and/or neuroinflammatory mechanisms^5–10^. Clozapine’s efficacy in TRS is likely mediated by its affinity at multiple receptors^11^. While the downstream neurobiological mechanisms are unclear, there is some evidence that clozapine modulation of glutamatergic^12^ and astrocyte activity^13^ or corticostriatal functional connectivity^14^ may be involved.

Regional cerebral blood flow (CBF), the rate at which blood is delivered to tissue, is intimately related to neural activity and metabolism by virtue of the phenomenon of neuro-vascular coupling^15^. Schizophrenia spectrum disorders are associated with CBF decreases in fronto-limbic areas and increases in subcortical areas^16^, which are associated with the severity of negative or positive symptoms, respectively. In addition, longitudinal studies show that antipsychotic treatment exerts regional effects on CBF, including increases in the basal ganglia and changes in frontal perfusion, which may vary between different antipsychotic medications^17^.

Single photon emission computed tomography (SPECT) and positron emission tomography (PET) studies have indicated the potential of CBF imaging at rest to detect changes in brain activity that occur in TRS and during clozapine treatment, as well as their associations with symptom improvement^18–24^. SPECT studies report widespread reductions in regional CBF across frontal (‘hypofrontality’), temporal and parietal cortices, the thalamus and striatum in patients with schizophrenia prior to commencing clozapine treatment^18,19,21,22^. Following clozapine administration, some SPECT/PET studies have reported CBF increases in frontal cortical and striatal regions, although there are mixed findings^18,20–24^.

In addition to methodological differences, it is possible that relationships between changes in CBF and the degree of clinical response to clozapine could explain some of the different results regarding changes in CBF after clozapine treatment^18,20–24^. For example, decreases in CBF in the striatum and thalamus^18^ or increases in frontal/caudate perfusion ratio^23^ after clozapine treatment have been reported in clozapine responders only. Furthermore, there is some indication that CBF before clozapine initiation may be predictive of subsequent response^18,20,23^. Compared to non-responders, clozapine responders may show higher CBF in the thalamus, left basal ganglia, and right prefrontal cortex prior to clozapine treatment^18,20^ and the right frontal/thalamus perfusion ratio before clozapine initiation may positively correlate with subsequent symptom improvement^23^. Changes in CBF also appear to be more extensive following treatment with clozapine compared to other antipsychotics^21,24^, suggesting that the extent of CBF change might relate to the superior efficacy of clozapine in TRS.

These seminal SPECT and PET studies of clozapine effects on CBF^18–24^ were published over almost 20 years ago. A more recent approach to regional CBF measurement is arterial spin labelling (ASL), which is a contrast‐free magnetic resonance imaging (MRI) technique that can detect changes in CBF with higher spatial resolution in a relatively short scan time and without the constraints of radiotracer administration^25^. As such, ASL may be easier to implement in a clinical setting or where repeated measurements are required. A recent comprehensive synthesis of MRI-based CBF studies (with the majority using ASL) reported consistently lower cortical CBF in schizophrenia spectrum disorders compared to in healthy volunteers, which was most apparent in the superior and middle frontal gyri, and higher CBF in the putamen^16^. Hypoperfusion in cortical fronto-temporal areas was related to the severity of negative symptoms^16,21^, whereas hyperperfusion in subcortical structures was related to positive symptom severity^16^. However, ASL has not yet been applied to investigate differences in CBF in TRS or changes in CBF during clozapine treatment.

In the present study, we used ASL to examine resting CBF in patients with TRS prior to commencing clozapine treatment and the changes in CBF that occur in TRS during clozapine treatment. Based on previous literature^16,18–24,26^, we selected four regions of interest (ROIs): the anterior cingulate (ACC) region of the frontal cortex, hippocampus, thalamus and striatum, and supplemented this with whole-brain analyses. We hypothesised that before starting clozapine, TRS would be associated with hypoperfusion in these ROIs, and that this would be normalised during clozapine treatment in parallel with symptomatic response. We additionally tested the hypothesis that higher regional CBF prior to commencing clozapine treatment would predict greater subsequent symptom improvement.

## Results

Data were available at baseline in 36 patients with TRS and 16 healthy controls. Data were available in 24 patients at follow-up after 12 weeks of clozapine treatment (Table 1). Of the 12 who discontinued the study, 2 never commenced clozapine, 3 discontinued clozapine due to adverse effects, 4 declined further MRI and 3 moved out of the area. There were no significant clinical or demographic differences between patients who did or did not complete the study (Table S1). The healthy volunteers were younger than the TRS group (*t*(50) = 2.25, *p* = 0.03), while sex did not differ by group (*p* > 0.05).

**Table 1:** Descriptive statistics of the patient sample at baseline and follow-up.

	Baseline	Week 12 follow-up
	*N* = 36	*N* = 24
Age, years	39.47 (13.82)	38.83 (13.04)
Sex, male/female	27/9	18/6
Age of onset, years	26.11 (9.00)	26.13 (8.68)
Duration of illness, years	14.31 (8.77)	13.92 (8.99)
Previous clozapine use, Yes/No	7/29	4/20
Diagnosis, F20-schizophrenia/F25-schizoaffective	30/6	21/3
	***N*** = **35**	
Previous antipsychotic trials, min; max; median	2; 10; 3	2; 7; 3
Number of hospital admissions, min; max; median	0; 12; 3	0; 12; 3
Symptoms and Functioning		
PANSS-Positive	18.43 (5.87)	14.33 (5.10)
PANSS-Negative	19.23 (7.51)	15.29 (5.57)
PANSS-General	35.33 (6.94)	27.04 (5.98)
PANSS-Total	72.97 (14.62)	56.29 (14.11)
GAF	45.29 (10.51)	59.42 (8.66)
	***N*** = **34**	
Antipsychotic dose, CPZE/mg per day	229.21 (187.44)	/
Clozapine		***N*** = **22**
Clozapine dose, daily mg	/	362.50 (140.31)
Plasma clozapine, ng/ml	/	473.18 (30.99)
Plasma norclozapine, ng/ml	/	240.00 (123.10)

Data shown as mean (standard deviation) unless otherwise specified.

*CPZE* chlorpromazine equivalent dose, *GAF* global assessment of functioning, *PANSS* positive and negative syndrome scale.

### CBF in TRS compared to healthy volunteers

#### ROI analysis

**Fig. 1:**
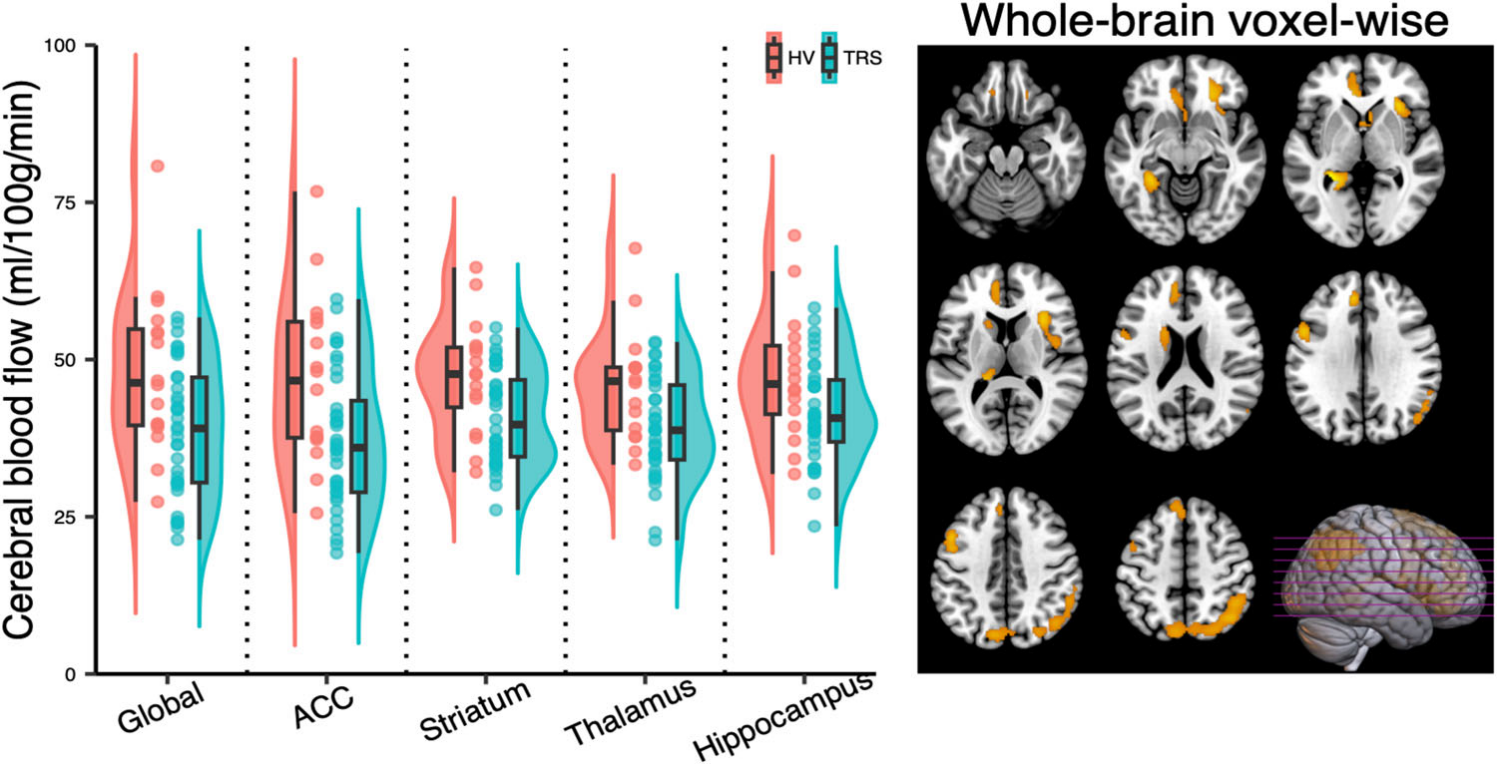
Differences in cerebral blood flow in treatment-resistant schizophrenia prior to clozapine initiation (*N* = 36) compared to healthy volunteers (*N* = 16). Left panel: Global and region of interest (ROI) data showing significantly lower global and regional cerebral blood flow (CBF) in the TRS compared to the HV group (*p* < 0.05), without controlling for age or global CBF. Half-violin plots depict the distribution of mean CBF values within each group, boxplots show median and interquartile ranges and circles provide individual values. Right panel: Brain sections illustrating the significant clusters (*p* < 0.05 family-wise error corrected) of lower CBF in the TRS compared to the HV group, controlling for age and global CBF. ACC anterior cingulate cortex, HV healthy volunteer, TRS treatment-resistant schizophrenia.

CBF was lower in the TRS group than in the healthy volunteer group across all ROI (ACC: *F*(1, 50) = 7.60, *p* = 0.01; Striatum: *F*(1, 50) = 7.16, *p* = 0.01; Thalamus: *F*(1, 50) = 7.50, *p* = 0.01; Hippocampus: *F*(1, 50) = 4.82, *p* = 0.03; Table 2; Fig. 1). However, global CBF was also lower in the TRS group (*F*(1, 50) = 7.46, *p* = 0.01). When age and global CBF were included as covariates in the model, there were no significant group differences in CBF across ROIs (all *p* > 0.05; Table 1).

**Table 2:** Regional cerebral blood flow in treatment-resistant schizophrenia prior to clozapine initiation and healthy volunteers.

	TRS	HV	No covariates				Covarying for age & global CBF		
	*N* = 36	*N* = 16	Test statistics				Test statistics		
			*F*		*df*	*P*	*F*	*df*	*P*
Global CBF, ml/100 g/min	38.83 (10.45)	48.08 (12.99)	7.46		1	0.01	/	/	/
Regional CBF, ml/100 g/min									
ACC	37.10 (10.96)	46.88 (13.56)	7.60	1		0.01	0.00	1	0.97
Striatum	40.83 (7.64)	47.36 (9.16)	7.16	1		0.01	0.44	1	0.51
Thalamus	39.04 (8.08)	45.91 (8.94)	7.50	1		0.01	0.66	1	0.42
Hipp	41.61 (8.08)	47.39 (10.15)	4.82	1		0.03	0.00	1	1.00

Data were presented as mean (standard deviation).

*ACC* anterior cingulate cortex, *CBF* cerebral blood flow, *Hipp* hippocampus, *HV* healthy volunteer, *TRS* treatment-resistant schizophrenia.

#### Whole-brain voxel-wise analysis

After controlling for age and global CBF, the voxel-wise comparison revealed significantly lower CBF in the TRS group than in healthy volunteers in several areas (Table S2 and Fig. 1), including the frontal (three clusters: MNI (x, y, z) = −8, 38, 30, *p* = 0.00 FWE; MNI (x, y, z) = −20, 22, 58, *p* = 0.02 FWE; MNI (x, y, z) = −46, 12, 40, *p* = 0.03 FWE), insula (one cluster: MNI (x, y, z) = 32, 22, 2, *p* = 0.01 FWE), parietal (one cluster: MNI (x, y, z) = 40, −62, 48, *p* = 0.00 FWE) cortices, and striatum (one cluster: MNI (x, y, z) = −26, −42, 2, *p* = 0.01 FWE). The opposite contrast (i.e., CBF in the TRS group > Healthy Volunteer group) revealed no significant clusters.

### Changes in CBF during clozapine treatment

#### ROI analysis

**Fig. 2:**
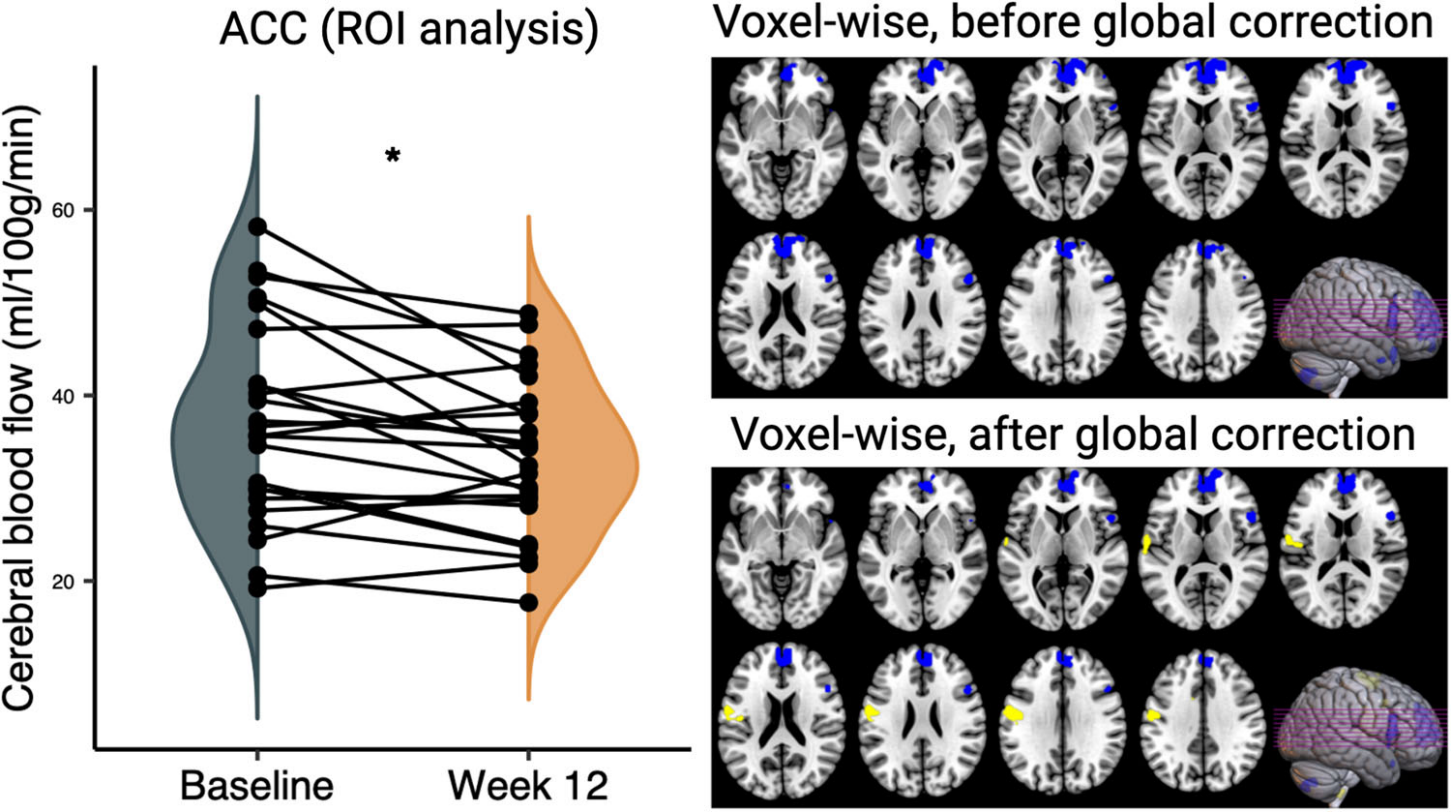
Changes in cerebral blood flow in treatment-resistant schizophrenia after 12 weeks of clozapine treatment (*N* = 24). Left panel: Region of interest (ROI) analysis showing decreased regional CBF in the ACC after 12 weeks of clozapine treatment (*p = 0.01). Half-violin plots depict the distribution of mean ACC CBF at each time point. Circles represent individual values. Right panel: Brain sections illustrating significant clusters (*p* < 0.05 family-wise error corrected) in whole-brain voxel-wise analyses before (top panel) or after (bottom panel) controlling for changes in global CBF. In both cases, decreased CBF (blue) following clozapine treatment was apparent in a cluster centred on the ACC. After controlling for CBF (bottom panel), a cluster of increased CBF following clozapine treatment was apparent in the left postcentral area (yellow). ACC anterior cingulate cortex, CBF cerebral blood flow, ROI region of interest.

Analysis of change in CBF during clozapine treatment with a Greenhouse-Geisser correction found a significant main effect of ROI (*F*(2.02, 46.49) = 21.13, *p* < 0.001) and significant Time * ROI interaction (*F*(1.67, 38.37) = 5.06, *p* = 0.02). These effects remained significant when covarying for change in global CBF (main effect of ROI: *F* (2.04, 44.92) = 21.96, *p* < 0.001; Time * ROI interaction: *F* (1.56, 34.30) = 4.39, *p* = 0.03). Subsequent repeated measures ANOVA in each ROI separately found CBF was significantly decreased in the ACC after 12 weeks of clozapine treatment compared to at baseline (*F*(1, 23) = 8.66, *p* = 0.01; Fig. 2 and Table 3), which remained significant when covarying for the numerical differences in global CBF over time (*F*(1, 22) = 11.18, *p* = 0.00; Table 3). There were no significant changes in CBF in the hippocampus, striatum, and thalamus or globally during clozapine treatment (all *p* > 0.05; Table 3).

**Table 3:** Regional cerebral blood flow before and after 12 weeks of clozapine treatment.

	Patient sample (*N* = 24)		No covariates			Covarying for percent change of global CBF		
	Baseline	Week 12	Test statistics			Test statistics		
			*F*	*df*	*P*	*F*	*df*	*P*
Global CBF, ml/100 g/min	38.62 (10.48)	36.26 (8.64)	3.00	1	0.10	/	/	/
Regional CBF, ml/100 g/min								
ACC	37.12 (10.72)	33.37 (8.35)	8.66	1	0.01	11.18	1	0.00
Striatum	40.43 (7.37)	39.44 (7.49)	0.76	1	0.39	0.07	1	0.80
Thalamus	38.55 (8.68)	36.87 (8.31)	1.61	1	0.22	0.04	1	0.84
Hipp	42.01 (8.01)	41.34 (7.98)	0.25	1	0.62	1.44	1	0.24
PANSS-Positive	18.92 (5.98)	14.33 (5.10)	73.47	1	<0.001	/	/	/
PANSS-Negative	18.13 (6.42)	15.29 (5.57)	8.84	1	0.01	/	/	/
PANSS-General	34.54 (7.44)	27.04 (5.98)	38.15	1	<0.001	/	/	/
PANSS-Total	71.58 (15.99)	56.29 (14.11)	58.54	1	<0.001	/	/	/
GAF	48.25 (9.31)	59.42 (8.66)	29.00	1	<0.001	/	/	/

Data were presented as mean (standard deviation).

*ACC* anterior cingulate cortex, *CBF* cerebral blood flow, *GAF* global assessment of functioning, *Hipp* hippocampus, *PANSS* positive and negative syndrome scale.

#### Whole-brain voxel-wise analysis

Whole-brain voxel-wise analysis (Table S3) revealed significantly decreased CBF in the medial frontal gyrus in the TRS group after 12 weeks of clozapine treatment (one cluster: MNI (x, y, z) = −2, 54, 30, *p* = 0.01 FWE; Fig. 2). Decreases in CBF in the medial frontal cortex were also significant when controlling for changes in global CBF (one cluster: MNI (x, y, z) = −2, 52, 30, *p* = 0.00 FWE; Fig. 2). The opposite contrast (i.e., CBF at Baseline < Week 12 follow-up) without covarying with changes in global CBF did not reveal any significant clusters. After controlling for changes in global CBF, this contrast revealed significantly increased CBF following clozapine treatment compared to baseline in the left postcentral gyrus (one cluster: MNI (x, y, z) = −58, −6, 22, *p* = 0.02 FWE; Fig. 2).

### Relationships between change in CBF and symptom improvement

Symptom severity scores for all PANSS (sub)scales were significantly lower after 12 weeks of clozapine treatment compared to the baseline values (Table 3).

The change in regional CBF in the ACC and thalamus was significantly (*p* < 0.05 uncorrected) associated with the percentage change in symptom scores over 12 weeks of clozapine treatment (Table S4). Specifically, greater reductions in ACC CBF were associated with lesser improvement in negative symptom scores (*r* = −0.49, df = 21, *p* = 0.01, covarying for change in global CBF; Fig. S1) and greater reductions in thalamus CBF were associated with lesser improvement in positive (*r* = −0.42, df = 21, *p* = 0.05, covarying for change in global CBF; Fig. S2) and total (*r* = −0.49, df = 21, *p* = 0.02, covarying for change in global CBF; Fig. S2) symptom scores. There were no significant relationships between plasma clozapine level or clozapine dose at 12 weeks and the percent change in CBF (all *p* > 0.05). Voxel-wise analyses did not detect any significant associations between the change in CBF and symptom scores over 12 weeks, plasma clozapine levels or clozapine dose.

### Relationship between CBF at baseline and symptom improvement

Baseline CBF in the striatum and hippocampus were significantly (*p* < 0.05 uncorrected) associated with subsequent percentage change in symptom scores (Table S5), such that participants with higher baseline striatum CBF had greater improvements in general symptom scores (*r* = 0.40, df = 25, *p* = 0.04, covarying for baseline global CBF; Fig. S3), and participants with higher baseline hippocampus CBF had greater improvements in total (*r* = 0.50, df = 25, *p* = 0.01, covarying for baseline global CBF; Fig. S4) and positive (*r* = 0.40, df = 25, *p* = 0.04, covarying for baseline global CBF; Fig. S4) symptom scores. There were no significant relationships between CPZE and regional CBF at baseline (all *p* > 0.05). No further significant correlations between ROI CBF and clinical variables were detected, and voxel-wise analyses did not find significant associations between baseline CBF and clinical variables.

### Grey matter volume

#### GMV in TRS compared to in healthy volunteers

Whole-brain GMV (Table S6) in the TRS group was significantly lower than in the healthy volunteer group (t(50) = −0.79, *p* = 0.01; Fig. S5). After controlling for age, sex and TIV, this effect remained significant (F(1) = 8.88, *p* = 0.01). The voxel-wise comparison revealed significantly lower global GMV in the TRS group than in healthy volunteers, with peaks in the right middle cingulate cortex and bilateral hippocampus (Table S2 and Fig. S5). The opposite contrast (i.e., TRS group > Healthy Volunteer group) revealed no significant clusters.

#### Changes in GMV during clozapine treatment

Within the TRS group, whole-brain GMV (Table S7) was significantly decreased after 12 weeks of clozapine treatment compared to baseline (*t*(23) = 4.04, *p* < 0.001; Fig. S6). Voxel-wise analysis revealed decreases in GMV across several areas, including in the frontal, temporal, parietal and occipital cortices, striatum and cerebellum (Table S3 and Fig. S6). The opposite contrast (i.e., Baseline < Week 12 follow-up) revealed no significant clusters.

#### Effect of GMV on CBF

Reductions in CBF in TRS compared to healthy volunteers were similar after controlling for GMV, although the group difference was no longer detected in the striatum (Table S2 and Fig. S7). Reductions in CBF during clozapine treatment in frontal regions were also apparent after controlling for change in GMV, but the increase in CBF in the left postcentral area was no longer significant (Table S3 and Fig. S7).

## Discussion

This study applied the MRI-based approach of pCASL to examine resting CBF in patients with TRS and the changes in CBF that occur in TRS during clozapine treatment. We found that CBF was lower in patients with TRS than in healthy volunteers across whole-brain grey matter and in the ACC, striatum, thalamus and hippocampus. After correction for global perfusion differences, analysis indicated these decreases were greatest in frontal and parietal regions and the left caudate. Contrary to our hypothesis that clozapine treatment would improve frontal cortical hypoperfusion in TRS, both the ROI and voxel-wise analyses found a further decrease in CBF in frontal regions, including the ACC after clozapine treatment. Although symptom severity overall improved with clozapine treatment, greater reductions in ACC CBF were associated with less improvement in negative symptoms, while greater reductions in CBF in the thalamus were associated with less improvement in positive and total symptoms. Furthermore, we found that higher CBF in the striatum and hippocampus at baseline predicted greater improvement in symptoms after clozapine treatment.

Our first finding that TRS is associated with widespread reductions in CBF is overall consistent with previous studies in TRS populations using SPECT methods^18,19,21,22^. The pattern of cortical hypoperfusion observed in TRS is also consistent with a previous synthesis of MRI studies examining CBF in non-TRS cohorts^16^. This suggests that cortical hypoperfusion is common across the schizophrenia spectrum, although it could be potentially more marked in TRS, as has been observed for cortical glucose hypometabolism^27^ and cortical structural deficits^28,29^. In contrast to findings in non-treatment-resistant schizophrenia that perfusion is increased in subcortical structures, including the putamen and thalamus^16^, we detected CBF decreases in subcortical regions in our TRS sample. The suggestions that TRS is associated with subcortical hypo- compared to hyper-perfusion and more marked cortical hypoperfusion should be further examined through direct comparison of TRS and non-TRS cohorts.

The decreases in frontal cortical (ACC) CBF that we observed after clozapine treatment differ from the previous observations in PET and SPECT studies that clozapine generally increases cortical perfusion^21,23,24^. However, other studies have reported that clozapine treatment is associated with CBF decreases in the ventrolateral and superior frontal cortices^24^, no change in resting CBF^22^, more extensive prefrontal perfusion deficits during task performance^30^ or decreased metabolism in the prefrontal cortices as observed with [^18^F]fluoro-deoxy-glucose (FDG)-PET^31^. These mixed findings may be attributed to several factors, including the different methods used to measure CBF and metabolism, durations of clozapine treatment as well as patient characteristics (including the presence of treatment resistance), all making it difficult to draw direct comparisons. One difference is that, in studies reporting increases or no change in cortical CBF over clozapine treatment^21–24^, the baseline scan was performed in the absence of antipsychotics, whereas in our study and those of Molina et al.^30^, patients were medicated at baseline and had been for several years. Furthermore, we also found that reductions in CBF in the ACC and thalamus were most apparent in patients with less negative symptom improvement, suggesting that decreases in CBF may correlate with poor negative symptom response to clozapine. Overall, these findings suggest that clozapine may worsen pre-existing ACC hypoperfusion in TRS rather than improve it. One explanation could be that antipsychotics, including clozapine place additional metabolic demands on brain tissue, including CBF, which can not be sustainably met^32^. In Alzheimer’s Disease, decreases in CBF may be associated with cognitive decline^33^, but the relationship between CBF and cognition in schizophrenia is unknown, and we did not measure cognition in our cohort. A recent meta-analysis found that clozapine does not have beneficial effects on cognitive function in TRS^34^, and a cross-sectional study indicated that higher doses of clozapine were associated with worse cognitive function^35^. This raises the possibility that decreases in frontal CBF during clozapine treatment may be related to cognition, which would be of interest to examine in future work.

We also found that higher CBF in the hippocampus and striatum before clozapine initiation was associated with greater subsequent symptom improvement. Similarly, previous studies reported higher baseline CBF in subcortical and cortical regions in clozapine responders compared to non-responders^18,20^, and that a higher baseline right frontal/thalamus perfusion ratio correlated with greater symptom improvement^23^. While these associations should be interpreted cautiously as they were not corrected for multiple comparisons, this could indicate that less marked CBF pathophysiology, indicative of more metabolic resource^32^ may facilitate clinical response to clozapine treatment.

TRS was also associated with reductions in GMV globally and in the ACC, thalamus and hippocampus, as overall consistent with previous observations^36–40^. During clozapine treatment, voxel-wise analyses detected further GMV decreases in cortical regions and the striatum, corresponding to the decreases in striatal volume and cortical thinning previously reported in the same cohort^41^. The patterns of reduction in CBF and GMV in TRS compared to healthy volunteers and during clozapine treatment were partially overlapping, however GMV reductions did not appear to fully explain the reductions in CBF, as cortical CBF alterations remained similar after correction for GMV alterations, which is similar to previous findings in schizophrenia^42^. In TRS, both CBF and GMV reductions were apparent in the ACC, but CBF alterations extended to wider cortical regions, and GMV reductions were apparent in the thalamus and hippocampus. After clozapine treatment, both CBF and GMV were reduced in frontal regions, but GMV reductions were more widespread, being apparent in further cortical regions and in the striatum and cerebellum. Both overlapping and differential patterns of functional and structural changes have previously been reported in schizophrenia compared to healthy volunteers^42^, where, similar to our study, overlapping alterations were most apparent in the ACC. However, ACC GMV reductions have only a modest effect on reductions in ACC CBF, suggesting that metabolic compensation may occur in functionally intact neurons^43^. The causative relationships between vascular, metabolic and neuronal structural alterations are unknown and may differ between brain regions. Our data tentatively suggests that after clozapine initiation in TRS, functional changes in cortical areas are accompanied by further structural alterations, whereas in the striatum, grey matter loss is not accompanied by CBF reduction, which could reflect functional compensation.

While, to our knowledge, this is the first study to apply ASL to examine CBF in TRS and during subsequent clozapine treatment, our study has some limitations and raises questions which can be addressed in future research. As we compared participants with TRS to healthy volunteers but not to a treatment-responsive schizophrenia group, we cannot directly infer to what extent CBF differences relate to treatment-resistant illness specifically. Secondly, as clozapine is the only recommended antipsychotic for TRS, we did not compare CBF changes during clozapine treatment to those observed following treatment with a non-clozapine antipsychotic, therefore we do not know whether clozapine produces distinct or similar CBF effects to other antipsychotic compounds. Similarly, this comparison would be required to confirm whether the prediction of clinical response by baseline CBF is clozapine-specific, or generalises to other antipsychotics, or is overall prognostic of outcome. Compared to radiotracer methods, one advantage of ASL is that data can be acquired repeatedly in the same individual. It would be of interest to examine CBF changes over a longer period of clozapine administration, in relation to cognitive function, as well as whether early changes in CBF may be predictive of longer-term outcomes.

In summary, our findings suggest that clozapine treatment may worsen the CBF deficits that are observed in patients with TRS who have been previously treated with non-clozapine antipsychotics, and that patients with less marked CBF reduction prior to clozapine initiation may be more likely to respond to it. In the context of the suggestion that, in some patients, long-term antipsychotic treatment may place unsustainable metabolic demands on the brain^32^, our findings indicate that clozapine may be most effective when metabolic capacity is more preserved. This mechanism may help explain why clozapine is more effective when initiated early in illness^44^. Future investigations of functional and structural changes in the same individuals may provide further information on the underlying neurobiological mechanisms, and may have the potential to identify patient subgroups most likely to respond to clozapine, or those who may benefit from adjunctive therapies.

## Materials and methods

### Participants and study design

This study was approved by the London South East National Health Service Research Ethics Committee (Reference 13/LO/1857). Changes in brain glutamate^12^, as well as cortical thickness and subcortical grey matter volume^41^ occurring in the same cohort of TRS participants during clozapine treatment have been reported previously.

Individuals with TRS ≥18 years old who met ICD-10 criteria for schizophrenia (F20) or schizoaffective disorder (F25), were recruited from inpatient and outpatient services in South London and Maudsley and Oxleas NHS Foundation Trusts. Participants with TRS who had the capacity to do so provided their written informed consent to participate, and the study was also open to participants lacking the mental capacity to consent, in which case a carer was advised on their behalf. The inclusion criteria stipulated that patients with TRS at baseline (a) were clozapine naïve or had not taken clozapine for ≥3 months prior to this study; (b) had ≥2 previous trials of a non-clozapine antipsychotic, each within the recommended dose range for ≥6 weeks^45^; (c) were being referred by their treating psychiatrist for clozapine initiation. There was no minimum level of symptom severity required for inclusion. Healthy volunteers were ≥18 years old, recruited from the same geographic area, and all provided written informed consent. Exclusion criteria for all participants included drug dependency according to DSM-IV, pregnancy, and the presence of contraindications to MRI at 3 Tesla. Previous ASL power analyses indicate sample sizes of 11–20 participants are required to detect a 10–15% within-subjects change in CBF with 90–95% power^46,47^.

Patients underwent clinical interviews and MRI scans at baseline (−14 to 0 days before clozapine titration) and again 12 weeks after clozapine initiation. This period was selected as consensus guidelines recommend 12 weeks as the minimum treatment period over which to evaluate clozapine response^45^. Clozapine was prescribed according to normal clinical care. CBF at baseline was compared to that in healthy volunteers.

### Clinical interviews

In the TRS group, medical history was obtained by clinical interview and review of medical records. The severity of symptoms was assessed using the Positive and Negative Syndrome Scale (PANSS)^48^, and functioning was assessed using the Global Assessment of Functioning Scale^49^ at baseline and week 12. Clozapine dose, plasma clozapine and nor-clozapine levels were measured at weeks 6 and 12. The percentage change in PANSS total and subscale scores from baseline to week 12 were calculated after subtraction of minimum possible scores^50^.

### MRI acquisition and image pre-processing

Data were acquired on a 3 Tesla MR-750 MR scanner (GE Healthcare, Milwaukee, Wisconsin, USA). Whole-brain cerebral perfusion images were acquired using a 3D pseudo-continuous arterial spin labelling (pCASL) sequence, which uses a 3D Fast Spin Echo spiral multi-shot readout with the following parameters: label duration = 1800 ms, post labelling delay = 2025 ms, TE = 12 ms, TR = 5500 ms, ETL = 64, total acquisition time = 6 min. 3D T1-weighted images were acquired in the sagittal plane (slice thickness: 1.2 mm, number of slices: 196, field of view 270 mm) using a three-dimensional T1-weighted inversion recovery spoiled gradient-echo (IR-SPGR) sequence, with parameters based on the Alzheimer’s Disease Neuroimaging Initiative (ADNI-GO, http://adni.loni.usc.edu/) (repetition time: 7.31 ms; echo time: 3.02 ms, inversion time 400 ms, flip angle 11°, acquisition matrix 256 × 256 × 196 over a field of view of 270 × 270 × 235.2 mm).

CBF maps were computed in the scanner with a voxel size of 2 × 2 × 3 mm^3^ in accordance with the ASL consensus paper^51^. Spatial normalisation of the CBF maps (to MNI standard space) was performed using the automatic software for ASL processing (ASAP) toolbox^52^ running in statistical parametric mapping software (SPM-12, Functional Imaging Laboratory (FIL), Wellcome Centre for Human Neuroimaging, Institute of Neurology, University College London (UCL), UK, http://www.fil.ion.ucl.ac.uk/spm/ software/spm12/) (see Supplementary Methods for details) in preparation for region of Interest (ROI) and voxel-wise (whole brain) analysis.

The primary ROI approach analysed the mean CBF in the ACC, hippocampus, thalamus and striatum in each participant and time point. These regions were selected based on previous research, as provided in the introduction. For this analysis, ROI masks in these regions were defined using ‘WFU PickAtlas’ in SPM-12, and the mean CBF in each ROI was extracted in the ASAP toolbox. Similarly, mean CBF values were extracted from the group grey matter mask (‘global CBF’) to investigate and account for differences in global perfusion. This ROI-based approach was complemented with an examination of CBF differences across the whole-brain using voxel-wise analyses across all grey matter voxels. T1-weighted images were also used to investigate the potential overlap between differences in CBF and differences in grey matter volume (GMV), using the Computational Anatomy Toolbox (CAT12) within SPM-12^53^ (see Supplementary Methods).

### Statistical analysis

Differences in demographic and clinical variables between and within groups were tested using Chi-square tests or *t*-tests for categorical and continuous data, respectively, with a two-tailed alpha of 0.05. Continuous variables with a normal distribution are presented as mean and standard deviations.

For the ROI analysis, mean CBF values were analysed in Statistical Package for the Social Sciences (SPSS 29, IBM Corp., Armonk, NY). Between-group differences in rCBF at baseline were analysed using separate analyses of variance (ANOVAs) for each ROI, as multicollinearity between CBF in individual regions violated MANOVA assumptions. Subsequent analyses of covariance (ANCOVA) included age and global CBF as covariates. Within-group differences (TRS at baseline vs. week 12) were estimated using repeated measures ANOVA with ‘ROI’ and ‘Time’ as within-subject factors with/without change in global CBF as a covariate. Significant ROI by Time interactions were further investigated by repeated measures ANOVA in each region separately. Relationships between baseline CBF or change in CBF and continuous clinical variables were examined by partial correlation covarying for change in global CBF or global CBF at baseline. Statistical thresholds were set at a two-tailed significance level of *p* < 0.05 uncorrected.

Whole-brain voxel-wise CBF analysis was conducted in SPM-12. Significant clusters were assessed at the *p* < 0.05 family-wise error (FWE) threshold from a cluster-forming threshold of *p* < 0.001. Statistical maps for rejecting the null hypothesis of equal perfusion between the healthy volunteer group and TRS group at baseline were generated through a two-sample t-test analysis, with/without age, global CBF (and global GMV to test the effect of GMV on CBF) as covariates. Changes in CBF in the TRS group over 12 weeks of clozapine treatment were analysed using a voxel-wise paired *t*-test with/without global CBF (and change in GMV to test the effect of GMV on CBF) as covariates. To permit investigation of relationships between voxel-wise change in CBF and change in continuous clinical variables, we calculated CBF difference images for each participant (follow-up image – baseline image) using the image calculator (ImCalc) function in SPM-12. The correlations between baseline CBF or longitudinal CBF changes and clinical variables were calculated with multiple regression with/without adjusting for global CBF at baseline or global CBF changes, respectively.

To investigate the potential overlap between differences in CBF and differences in GMV, whole-brain GMV were extracted from CAT12 and input to SPSS. Between-group differences in whole-brain GMV were examined using one-way ANOVA and subsequent univariate linear models, with/without covarying for age, sex and TIV. Within-group differences in whole-brain GMV were estimated using paired-sample *t*-test. Statistical thresholds were set at a two-tailed significance level of *p* < 0.05. Voxel-wise comparisons of GMV were conducted in CAT12, SPM-12. Between-group differences at baseline were examined using a voxel-wise two-sample *t*-test analysis with two different contrasts (i.e., TRS group </> Healthy Volunteer group), adjusted for age, sex and TIV. Changes in GMV in the TRS group during clozapine treatment were analysed using a voxel-wise paired t-test with two different contrasts (i.e., Baseline </> Week 12 Follow-up). Significant clusters were assessed at the *p* < 0.05 FWE threshold from a cluster-forming threshold of *p* < 0.001.

## Supplementary information

Supplementary Material_Junyu 1120

## Data Availability

The anonymised data that support the findings of this study are available as open data via the King’s Open Research Data System (KORDS) at 10.18742/25664487.
