# Supplementary material for: Response to clozapine in treatment resistant schizophrenia is related to alterations in regional cerebral blood flow
Source: Schizophrenia (Heidelb). 2024 Dec 23;10(1):122. doi: 10.1038/s41537-024-00544-3 (PMC11666760; doi:10.1038/s41537-024-00544-3)
Supplement: Supplementary file 1 — Supplementary Material_Junyu 1120 [file 41537_2024_544_MOESM1_ESM.pdf]

Supplementary material

Response to clozapine in treatment resistant schizophrenia is related to alterations in regional cerebral blood flow

Junyu Sun*, Fernando Zelaya, Kyra-Verena Sendt, Grant McQueen, Amy L. Gillespie, John Lally, Oliver Howes, Gareth J. Barker, Philip McGuire, James H. MacCabe & Alice Egerton

SUPPLEMENTARY METHODS **2**

IMAGE PRE-PROCESSING 2

Pseudo-continuous arterial spin labelling (pCASL) *2*

Grey matter volume (GMV) *2*

SUPPLEMENTARY TABLES **3**

TABLE S1. BASELINE DEMOGRAPHIC AND CLINICAL VARIABLES OF PATIENTS WHO DID OR DID NOT COMPLETE FOLLOW-UP MRI AT 12-WEEKS 3

TABLE S2. VOXEL-WISE DIFFERENCES IN CEREBRAL BLOOD FLOW AND GREY MATTER VOLUME IN TREATMENT RESISTANT SCHIZOPHRENIA COMPARED TO HEALTHY VOLUNTEERS 4

TABLE S3. VOXEL-WISE DIFFERENCES IN CEREBRAL BLOOD FLOW AND GREY MATTER VOLUME IN TREATMENT RESISTANT SCHIZOPHRENIA BEFORE AND AFTER 12 WEEKS OF CLOZAPINE TREATMENT (N=24). 5

TABLE S4. RELATIONSHIPS BETWEEN CHANGES IN CEREBRAL BLOOD FLOW AND SYMPTOM IMPROVEMENT DURING CLOZAPINE TREATMENT. 6

TABLE S5. RELATIONSHIPS BETWEEN BASELINE CEREBRAL BLOOD FLOW AND SUBSEQUENT SYMPTOM IMPROVEMENT AFTER 12 WEEKS OF CLOZAPINE TREATMENT. 7

TABLE S6. WHOLE BRAIN STRUCTURAL VARIABLES IN TREATMENT RESISTANT SCHIZOPHRENIA COMPARED TO HEALTHY VOLUNTEERS 8

TABLE S7. WHOLE BRAIN STRUCTURAL VARIABLES IN TREATMENT RESISTANT SCHIZOPHRENIA BEFORE AND AFTER 12 WEEKS OF CLOZAPINE TREATMENT (N=24). 8

SUPPLEMENTARY FIGURES **9**

FIGURE S1. RELATIONSHIP BETWEEN CHANGES IN CEREBRAL BLOOD FLOW IN THE ANTERIOR CINGULATE CORTEX AND CHANGE IN NEGATIVE SYMPTOM SEVERITY OVER 12 WEEKS OF CLOZAPINE TREATMENT. 9

FIGURE S2. RELATIONSHIP BETWEEN CHANGES IN CEREBRAL BLOOD FLOW IN THE THALAMUS AND CHANGE IN POSITIVE (LEFT) AND TOTAL (RIGHT) SYMPTOM SEVERITY OVER 12 WEEKS OF CLOZAPINE TREATMENT. 9

FIGURE S3. RELATIONSHIP BETWEEN CEREBRAL BLOOD FLOW IN THE STRIATUM BEFORE COMMENCING CLOZAPINE TREATMENT AND THE SUBSEQUENT CHANGE IN GENERAL SYMPTOM SEVERITY OVER 12 WEEKS OF CLOZAPINE TREATMENT. 10

FIGURE S4. RELATIONSHIP BETWEEN CEREBRAL BLOOD FLOW IN THE HIPPOCAMPUS BEFORE COMMENCING CLOZAPINE TREATMENT AND THE SUBSEQUENT CHANGE IN TOTAL (LEFT) AND POSITIVE (RIGHT) SYMPTOM SEVERITY OVER 12 WEEKS OF CLOZAPINE TREATMENT. 10

FIGURE S5. DIFFERENCES IN GREY MATTER VOLUME IN TREATMENT RESISTANT SCHIZOPHRENIA COMPARED TO IN HEALTHY VOLUNTEERS 11

FIGURE S6. CHANGES IN GREY MATTER VOLUME IN TREATMENT RESISTANT SCHIZOPHRENIA AFTER 12 WEEKS OF CLOZAPINE TREATMENT. 12

FIGURE S7. CHANGES CEREBRAL BLOOD FLOW CONTROLLING FOR DIFFERENCES IN GREY MATTER VOLUME. 13

REFERENCES **14**

Supplementary Methods

Image pre-processing

Pseudo-continuous arterial spin labelling (pCASL)

Cerebral blood flow (CBF) maps were pre-processed using the standard pipeline in the Automatic Software for ASL Processing (ASAP) Toolbox[1]. First, the proton density images were co-registered to the high-resolution 3D T1-weighted image after realigning the origin of both images. The transformation parameters were then saved and applied to the CBF maps. The extracerebral signal from these CBF maps was eliminated via the multiplication of a binary mask generated by unified segmentation of the T1-weighted image. CBF maps were then normalized to MNI space by applying the previously saved unified segmentation registration parameters (T1 to MNI). Finally, normalized CBF maps were smoothed using an 8-mm full width at half maximum (FWHM) Gaussian kernel.

Grey matter volume (GMV)

Total and voxel-wise GMV were estimated using voxel-based Morphometry (VBM) analysis of the T1-weighted images using the Computational Anatomy Toolbox (CAT12), following the CAT12 manual, https://neuro-jena.github.io/cat//index.html[2] in SPM-12. T1-weighted images were reoriented and co-registered to the same point of origin and segmented to create grey matter (GM), white matter (WM), cerebrospinal fluid (CSF)[3], followed by spatial registration using the MNI standard space template as a reference[4]. For the within-group comparison, a longitudinal pipeline that considers deformations between individual images across time points was used to maximise sensitivity to effects. Quality control (single slice display and sample homogeneity) via the established framework[2] was employed to check data quality. Specifically, all images were visually inspected in CAT12 for potential segmentation and registration errors using single slice display, and checked for sample homogeneity by visualizing of the overall correlation of the images as a violin plot and correlation matrix, thus facilitating the identification of potential outliers. No images were excluded due to poor quality. The GM images were then smoothed using a Gaussian kernel of 8-mm FWHM. Total intracranial volume (TIV) was calculated for each subject to account for inter-individual differences in brain size. We extracted the mean values of whole brain GM, WM, CSF, and TIV for each image.

Supplementary Tables

Table S1. Baseline demographic and clinical variables of patients who did or did not complete follow-up MRI at 12-weeks.

**Baseline**	**Week 12 FP Completers N=24**	**Week 12 FP Non-completers N=12**	t/F	P
Age, years	38.83 (13.04)	40.75 (15.80)	0.39	0.70
Sex, Male/Female	18/6	9/3	0.00	1.00
Age of onset, years	26.13 (8.68)	26.08 (10.01)	-0.01	0.99
Duration of illness, years	13.92 (8.99)	15.08 (8.65)	0.37	0.71
Previous clozapine use, Yes/No	4/20	3/9	0.35	0.66
Diagnosis, F20-schizophrenia/F25-schizoaffective	21/3	9/3	0.90	0.38
Global CBF, ml/100g/min	38.62 (10.48)	39.24 (10.86)	0.16	0.87
Regional CBF, ml/100g/min				
ACC	37.12 (10.72)	37.07 (11.91)	-0.14	0.99
Striatum	40.43 (7.37)	41.62 (8.41)	0.44	0.67
Thalamus	38.55 (8.68)	40.01 (6.98)	0.51	0.62
Hippocampus	42.01 (8.01)	40.81 (8.53)	-0.42	0.68
		**N=11**		
Previous antipsychotic trials, min; max; median; range	2; 7; 3; 5	2; 10; 4; 8	3.75	0.71
Number of hospital admissions, min; max; median; range	0; 12; 3; 12	1; 12; 4; 11	12.19	0.30
Symptoms and Functioning				
PANSS-Positive	18.92 (5.98)	17.36 (5.73)	-0.72	0.48
PANSS-Negative	18.13 (6.42)	21.64 (9.37)	1.30	0.20
PANSS-General	34.54 (7.44)	36.92 (5.78)	0.97	0.34
PANSS-Total	71.58 (15.99)	76.00 (11.16)	0.83	0.42

Data shown as Mean (Standard Deviation) unless otherwise specified. ACC, Anterior Cingulate Cortex; CBF, Cerebral Blood Flow; FP, Follow-up; PANSS, Positive and Negative Syndrome Scale.

Table S2. Voxel-wise differences in cerebral blood flow and grey matter volume in treatment resistant schizophrenia compared to healthy volunteers.

Covariate	Contrast	Region	Anatomical Description	P	MNI Coordinates			Cluster Size
					x	y	z	
**Cerebral blood flow**								
**age & global CBF**	HV > TRS	Frontal	Left Superior Medial Frontal Gyrus	0.001	-8	38	30	1292
		Frontal	Left Superior Frontal Gyrus	0.021	-20	22	58	671
		Frontal	Left Middle Frontal Gyrus	0.034	-46	12	40	577
		Parietal	Right Angular Gyrus	0.000	40	-62	48	2168
		Insular	Right Insula	0.012	32	22	2	774
		Striatum	Left Caudate Nucleus	0.010	-26	-42	2	825
**age, global CBF & GMV**	HV > TRS	Frontal	Left Anterior Cingulate Cortex	0.035	-8	30	-4	565
		Parietal	Right Angular Gyrus	0.016	44	-48	50	713
		Insular	Right Insula	0.029	30	24	2	604
		Hippocampus	Left Hippocampus	0.014	-28	-42	-4	737
**Grey matter volume**								
**age, sex & TIV**	HV > TRS	Frontal	Right Middle Cingulate	0.018	6	0	39	1033
		Hippocampus	Right Hippocampus	0.000	26	-24	-18	2820
		Hippocampus	Left Hippocampus	0.000	-20	-34	6	2562

CBF: Cerebral Blood Flow; GMV: Grey Matter Volume; HV: Healthy Volunteer (N = 16); TIV: Total Intracranial Volume; TRS: Treatment Resistant Schizophrenia (N = 36).

Table S3. Voxel-wise differences in cerebral blood flow and grey matter volume in treatment resistant schizophrenia before and after 12 weeks of clozapine treatment (N=24).

Covariate	Contrast	Region	Anatomical Description	P	MNI Coordinates			Cluster Size
					x	y	z	
**Cerebral Blood Flow**								
**None**	BL > FP	Frontal	Left Superior Medial Frontal Gyrus	0.006	-2	54	30	2644
**global CBF**	BL > FP	Frontal	Left Superior Medial Frontal Gyrus	0.001	-2	52	30	1414
	BL < FP	Frontal	Left Postcentral Gyrus	0.017	-58	-6	22	697
**global CBF & GMV**	BL > FP	Frontal	Right Superior Medial Frontal Gyrus	0.000	8	48	34	3228
		Frontal	Right Middle Frontal Gyrus	0.036	42	20	38	550
**Grey Matter Volume**								
**None**	BL > FP	Frontal	Left Superior Medial Frontal Gyrus	0.000	-12	56	33	4628
		Frontal	Right Superior Frontal Gyrus	0.006	34	62	15	776
		Frontal	Right Inferior Frontal Gyrus	0.031	51	50	-3	544
		Temporal	Left Inferior Temporal Gyrus	0.000	-56	-39	-18	3086
		Temporal	Right Middle Temporal Gyrus	0.007	50	-26	-3	766
		Temporal	Left Superior Temporal Gyrus	0.017	-28	9	-20	629
		Parietal	Right Precuneus	0.000	10	-51	28	1244
		Parietal	Left Posterior Cingulate Gyrus	0.003	-8	-33	33	914
		Occipital	Left Calcarine Gyrus	0.005	-2	-81	4	821
		Striatum	Right Caudate	0.000	10	15	12	2808
		Striatum	Right Pallidum	0.000	21	0	4	2192
		Striatum	Left Putamen	0.000	-27	-8	3	1590
		Cerebellum	Left Lobule VI of Cerebellar Hemisphere	0.034	-34	-58	-33	533

BL, Baseline (prior to clozapine initiation); CBF, Cerebral Blood Flow; FP, Follow-up (after 12 weeks clozapine treatment); GMV, Grey Matter Volume.

Table S4. Relationships between changes in cerebral blood flow and symptom improvement during clozapine treatment.

**Percent change in CBF**	**Percent change in symptom severity scores**	**Test statistics**		
		r	N	P
**Global CBF**	PANSS-Positive	-0.211	24	0.321
	PANSS-Negative	0.235	24	0.268
	PANSS-General	0.349	24	0.094
	PANSS-Total	0.247	24	0.245
**Regional CBF**		**Controlling for percent change of global CBF**		
		r	df	P
ACC	PANSS-Positive	-0.138	21	0.530
	PANSS-Negative	-0.493	21	**0.017**
	PANSS-General	-0.036	21	0.871
	PANSS-Total	-0.252	21	0.246
Striatum	PANSS-Positive	-0.247	21	0.255
	PANSS-Negative	-0.021	21	0.924
	PANSS-General	-0.307	21	0.155
	PANSS-Total	-0.361	21	0.091
Thalamus	PANSS-Positive	-0.417	21	**0.048**
	PANSS-Negative	-0.269	21	0.214
	PANSS-General	-0.335	21	0.118
	PANSS-Total	-0.494	21	**0.017**
Hippocampus	PANSS-Positive	-0.291	21	0.179
	PANSS-Negative	-0.082	21	0.709
	PANSS-General	-0.190	21	0.385
	PANSS-Total	-0.300	21	0.165

ACC, Anterior Cingulate Cortex; CBF, Cerebral Blood Flow; PANSS, Positive and Negative Syndrome Scale. P values are uncorrected.

Table S5. Relationships between baseline cerebral blood flow and subsequent symptom improvement after 12 weeks of clozapine treatment.

Baseline CBF	Percent change in symptom severity scores	Test statistics		
		r	N	P
**Global CBF**	PANSS-Positive	-0.181	28	0.355
	PANSS-Negative	-0.227	28	0.245
	PANSS-General	-0.291	28	0.134
	PANSS-Total	-0.350	28	0.068
**Regional CBF**		**Controlling for baseline global CBF**		
		r	df	P
ACC	PANSS-Positive	0.126	25	0.530
	PANSS-Negative	-0.015	25	0.941
	PANSS-General	-0.074	25	0.713
	PANSS-Total	0.004	25	0.983
Striatum	PANSS-Positive	0.263	25	0.185
	PANSS-Negative	-0.217	25	0.278
	PANSS-General	0.403	25	**0.037**
	PANSS-Total	0.375	25	0.054
Thalamus	PANSS-Positive	0.273	25	0.168
	PANSS-Negative	0.124	25	0.538
	PANSS-General	0.087	25	0.668
	PANSS-Total	0.222	25	0.266
Hippocampus	PANSS-Positive	0.403	25	**0.037**
	PANSS-Negative	0.190	25	0.343
	PANSS-General	0.337	25	0.086
	PANSS-Total	0.496	25	**0.009**

ACC, Anterior Cingulate Cortex; CBF, Cerebral Blood Flow; PANSS, Positive and Negative Syndrome Scale. P values are uncorrected.

Table S6. Whole brain structural variables in treatment resistant schizophrenia compared to healthy volunteers.

	TRS group	HV group	Test statistics			Test statistics		
	N = 36	N=16	t	df	P	F	df	P
			Before controlling for age, sex & TIV			After controlling for age, sex & TIV		
**TIV, litres**	1.45 (0.16)	1.49 (0.13)	-0.792	50	0.432	/	/	/
GM volume, litres	0.66 (0.07)	0.72 (0.07)	-2.839	50	**0.007**	8.881	1	**0.005**
WM volume, litres	0.50 (0.07)	0.52 (0.06)	-0.756	50	0.453	0.343	1	0.561
CSF volume, litres	0.29 (0.07)	0.25 (0.04)	2.464	47.597	**0.017**	2.776	1	0.102

Values are presented as mean (standard deviation). CSF, Cerebrospinal Fluid; GM, Grey Matter; HV, Healthy Volunteer; TIV, Total Intracranial Volume; TRS, Treatment Resistant Schizophrenia; WM, White Matter. P values are uncorrected.

Table S7. Whole brain structural variables in treatment resistant schizophrenia before and after 12 weeks of clozapine treatment (N=24).

	Baseline	Week 12 Follow-up	Test statistics		
			t	df	P
**TIV, litres**	1.53 (0.15)	1.53 (0.15)	-0.153	23	0.880
GM volume, litres	0.66 (0.06)	0.64 (0.07)	4.043	23	**<0.001**
WM volume, litres	0.51 (0.07)	0.51 (0.07)	-0.894	23	0.380
CSF volume, litres	0.36 (0.05)	0.38 (0.06)	-3.073	23	**0.005**

Values are presented as mean (standard deviation). CSF, Cerebrospinal Fluid; GM, Grey Matter; TIV, Total Intracranial Volume; WM, White Matter. P values are uncorrected.

Supplementary Figures

Figure S1. Relationship between changes in cerebral blood flow in the anterior cingulate cortex and change in negative symptom severity over 12 weeks of clozapine treatment.

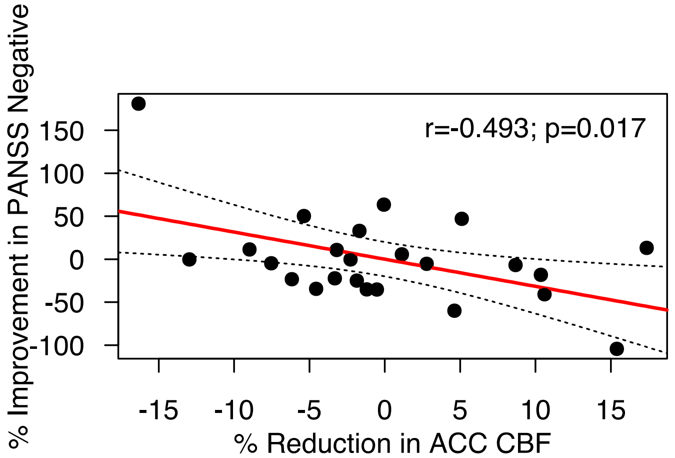

The scatter plot shows the relationship between percent change in cerebral blood flow in the anterior cingulate cortex and the percent change in negative symptoms over 12 weeks of clozapine treatment, controlling for global change in cerebral blood flow. The dashed lines present the 95% confidence intervals. ACC, Anterior Cingulate Cortex; CBF, Cerebral Blood Flow; PANSS, Positive and Negative Syndrome Scale.

Figure S2. Relationship between changes in cerebral blood flow in the thalamus and change in positive (left) and total (right) symptom severity over 12 weeks of clozapine treatment.

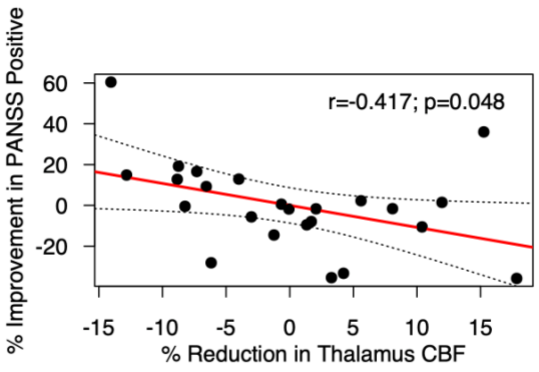

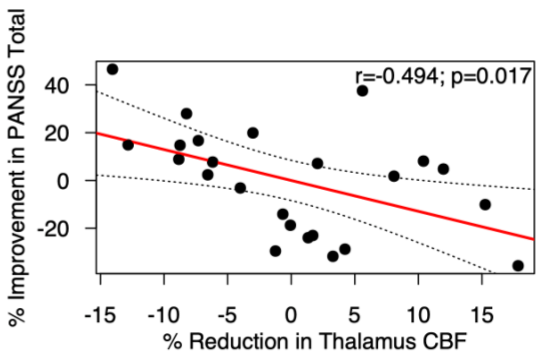

The scatter plots show the relationship between percent change in cerebral blood flow in the thalamus and the percent change in positive (left) and total (right) symptoms over 12 weeks of clozapine treatment, controlling for global change in cerebral blood flow. The dashed lines present the 95% confidence intervals. CBF, Cerebral Blood Flow; PANSS, Positive and Negative Syndrome Scale.

Figure S3. Relationship between cerebral blood flow in the striatum before commencing clozapine treatment and the subsequent change in general symptom severity over 12 weeks of clozapine treatment.

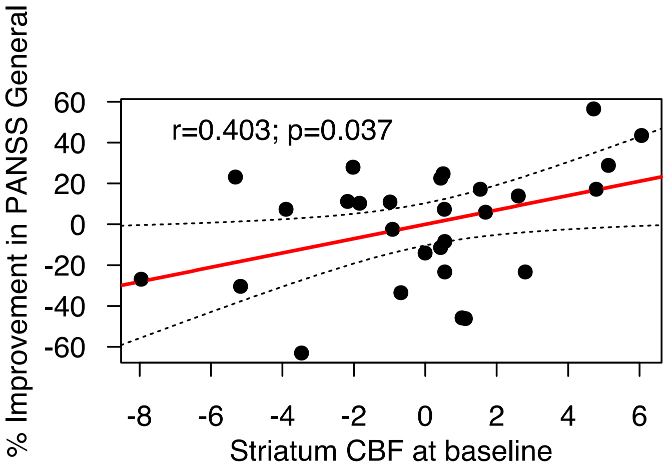

The scatter plot shows the relationship between percent change in cerebral blood flow in the striatum before commencing clozapine and subsequent percent change in general symptoms after 12 weeks of clozapine treatment, controlling for global change in cerebral blood flow. The dashed lines present the 95% confidence intervals. CBF, Cerebral Blood Flow; PANSS, Positive and Negative Syndrome Scale.

Figure S4. Relationship between cerebral blood flow in the hippocampus before commencing clozapine treatment and the subsequent change in total (left) and positive (right) symptom severity over 12 weeks of clozapine treatment.

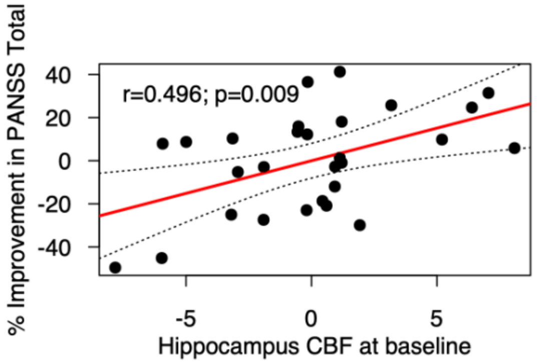

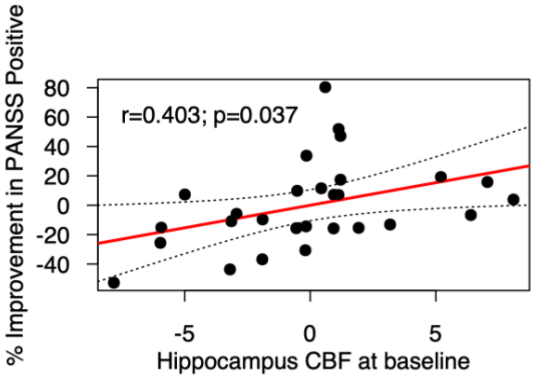

The scatter plots show the relationship between percent change in cerebral blood flow in the hippocampus before commencing clozapine and subsequent percent change in total (left) and positive (right) symptoms over 12 weeks of clozapine treatment, controlling for global change in cerebral blood flow. The dashed lines present the 95% confidence intervals. CBF, Cerebral Blood Flow; PANSS, Positive and Negative Syndrome Scale.

Figure S5. Differences in grey matter volume in Treatment Resistant Schizophrenia compared to in healthy volunteers.

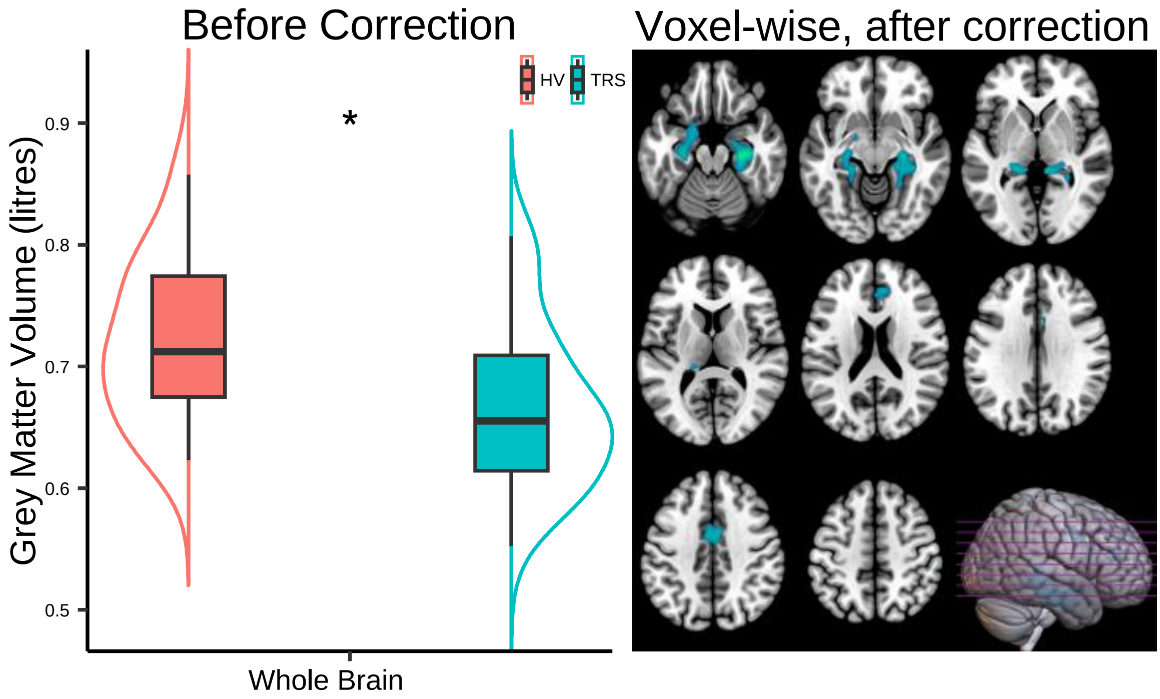

Left panel: Difference in total grey matter volume in Treatment Resistant Schizophrenia (N = 36) compared to healthy volunteers (N = 16) (* p=0.007), without controlling for age, sex or TIV. Half-violin plots depict the distribution of mean grey matter volume values within each group, boxplots show median and interquartile ranges and circles provide individual values.

Right panel: Brain sections illustrating the significant clusters (p < 0.05 family-wise error corrected) of lower grey matter volume in Treatment Resistant Schizophrenia (N = 36) compared to healthy volunteers (N = 16) controlling for age, sex and TIV.

HV, Healthy Volunteer; TRS, Treatment Resistant Schizophrenia; TIV, Total Intracranial Volume.

Figure S6. Changes in grey matter volume in Treatment Resistant Schizophrenia after 12 weeks of clozapine treatment.

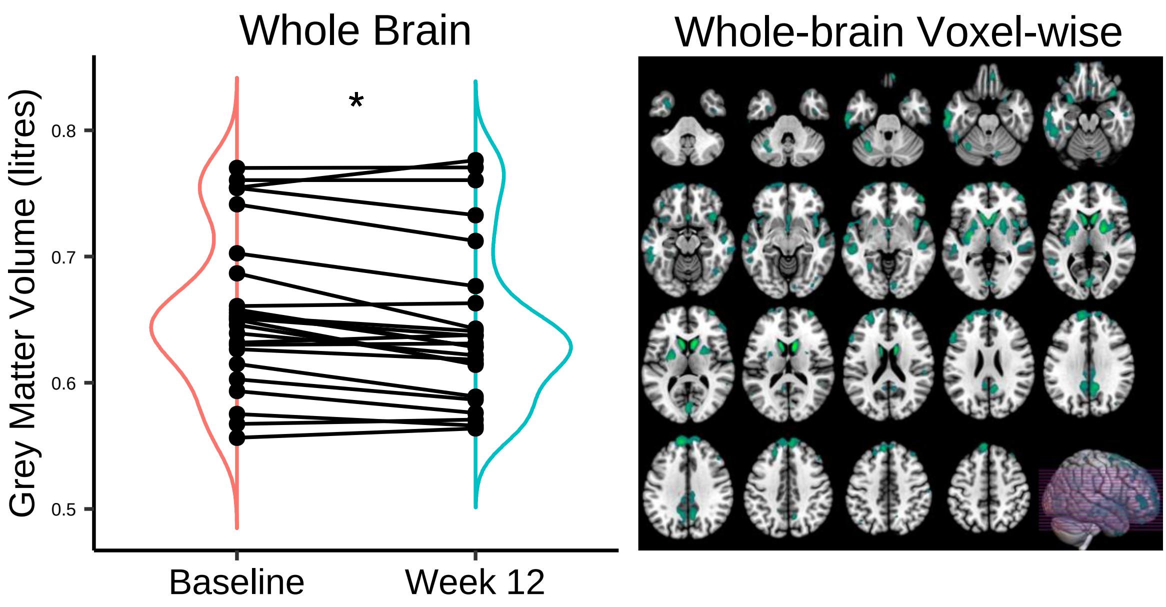

Left panel: Whole brain analysis showing decreased total grey matter volume after 12 weeks of clozapine treatment (* p<0.001). Half-violin plots depict the distribution of mean total grey matter volume at each time-point. Circles represent individual values.

Right panel: Brain sections illustrating clusters of significantly decreased grey matter (p < 0.05 family-wise error corrected) after 12 weeks of clozapine treatment.

Figure S7. Changes cerebral blood flow controlling for differences in grey matter volume.

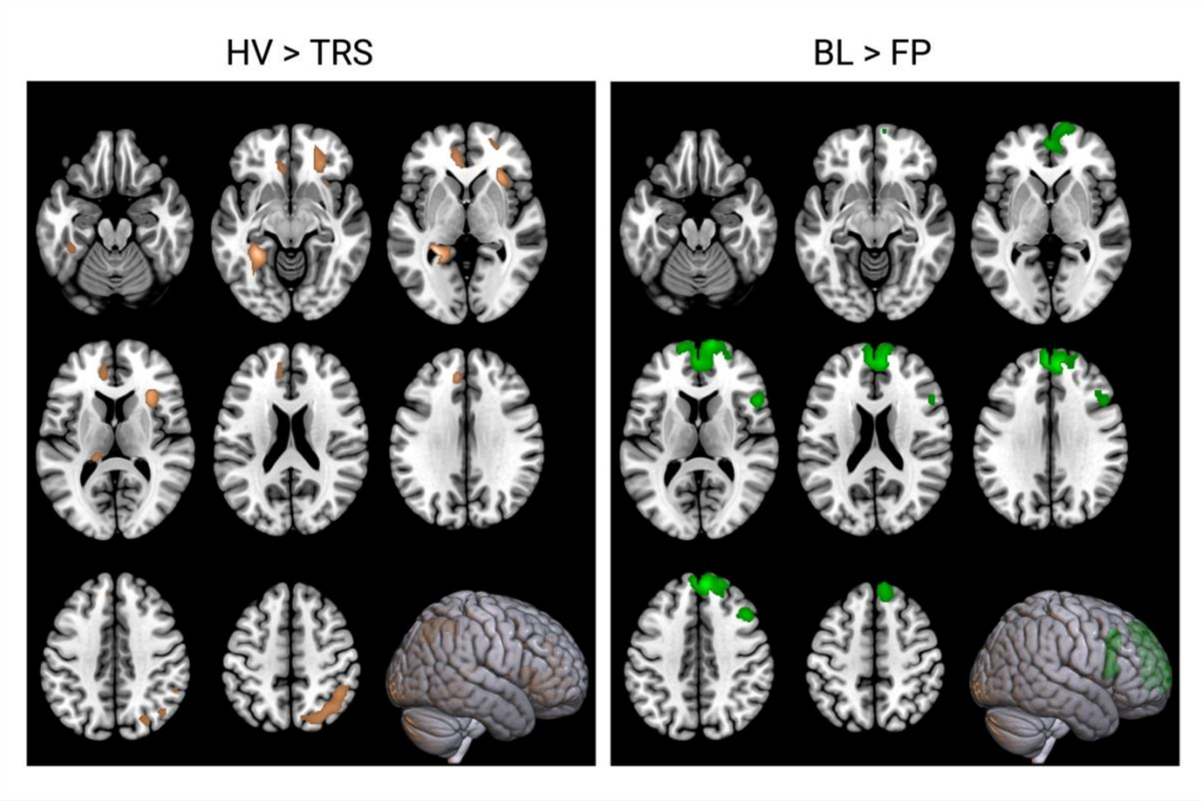

Left panel: Brain sections showing the significant clusters ($p < 0.05$ family-wise error corrected) of lower cerebral blood flow in treatment resistant schizophrenia prior to clozapine compared to in healthy volunteers, controlling for age, global cerebral blood flow & grey matter volume. HV, Healthy Volunteer; TRS, Treatment Resistant Schizophrenia.

Right panel: Brain sections illustrating significant clusters ($p < 0.05$ family-wise error corrected) of decreased cerebral blood flow after 12 weeks of clozapine treatment, controlling for changes in global cerebral blood flow & grey matter volume. BL, Baseline (prior to clozapine initiation); FP, week 12 Follow-up.

References

1 Mato Abad, V., Garcia-Polo, P., O'Daly, O., Hernandez-Tamames, J. A. & Zelaya, F. ASAP (Automatic Software for ASL Processing): A toolbox for processing Arterial Spin Labeling images. *Magn Reson Imaging* **34**, 334-344 (2016). https://doi.org/10.1016/j.mri.2015.11.002

2 Gaser, C., Dahnke, R., Thompson, P. M., Kurth, F. & Luders, E. CAT – A Computational Anatomy Toolbox for the Analysis of Structural MRI Data (2023). https://doi.org/10.1101/2022.06.11.495736

3 Ashburner, J. & Friston, K. J. Unified segmentation. *Neuroimage* **26**, 839-851 (2005). https://doi.org/10.1016/j.neuroimage.2005.02.018

4 Ashburner, J. A fast diffeomorphic image registration algorithm. *Neuroimage* **38**, 95-113 (2007). https://doi.org/10.1016/j.neuroimage.2007.07.007
